# The lineage diversity, spatiotemporal distribution and pathological significance of *Plasmodium* and *Haemoproteus* spp. infection of wild birds in Great Britain

**DOI:** 10.1016/j.ijppaw.2025.101148

**Published:** 2025-10-18

**Authors:** Joseph P. Heaver, Shinto K. John, Katharina Seilern-Macpherson, Simon Spiro, Vicky Wilkinson, Andrew A. Cunningham, Becki Lawson

**Affiliations:** a Institute of Zoology, Zoological Society of London, Regent's Park, London, NW1 4RY, UK; b Wildlife Health Services, Zoological Society of London, Regent's Park, London, NW1 4RY, UK

**Keywords:** Haemoparasites, Malaria, *Plasmodium*, *Haemoproteus*, PCR, Wild birds

## Abstract

Avian haemosporidian parasites (AHPs), which include the genera *Plasmodium* and *Haemoproteus*, are protist parasites affecting at least 2000 species of birds with near global distribution. Outside of isolated, evolutionarily and immunologically naïve avian populations, the effects of AHPs on wild bird populations are poorly understood but have historically been considered benign. There is growing evidence to suggest, however, that high exoerythrocytic parasite burdens can cause disease and mortality in some host-parasite interactions, even in populations which have co-evolved alongside AHPs. Here, samples from 857 wild birds of 62 species, 27 families and eight orders were collected during post-mortem examinations over a 15-year period as part of a nationwide wildlife disease surveillance scheme and were screened by nested polymerase chain reaction (PCR) for the presence of *Plasmodium* and *Haemoproteus*. In total, liver and/or spleen tissues from 13.5 % of birds (n = 116) tested PCR-positive, comprising 8.9 % (n = 76) and 4.7 % (n = 40) infected with *Plasmodium* and *Haemoproteus* spp., respectively. The highest rates of *Plasmodium* infection were seen in the families Paridae (36.3 %; 4/11 birds examined) and Turdidae (34.5 %; 51/148), consistent with previous reports. Spatial analysis revealed a significant cluster of *Plasmodium*-positive cases in Southeast England with possible explanations including climatic effects on parasite development or spatial variation in vector abundance. A total of 30 AHP lineages (20 *Haemoproteus* spp. and 10 *Plasmodium* spp.) were detected, 23 of which have not previously been reported in Great Britain, with four being apparently novel. Tissue samples from a subset of 13 *Plasmodium*-positive Eurasian blackbirds (*Turdus merula*) underwent histopathological examination, which revealed evidence of exoerythrocytic parasites, or other lesions consistent with avian malaria, in four and five cases, respectively. These changes were considered of equivocal significance in four birds, with only one bird diagnosed with acute malaria as a contributory cause of death.

## Highlights

• Tissue samples from 857 wild birds were screened for *Plasmodium* and *Haemoproteus*.
• 8.9 % of birds were infected with *Plasmodium* spp. and 4.7 % with *Haemoproteus* spp.
• Turdidae and Paridae species exhibited highest rates of *Plasmodium* infection.
• Of 30 AHP lineages detected, 23 are new to GB and four are apparently novel.
• Histopathology on 13 blackbirds detected avian malaria in a minority of cases.

## Graphical abstract

**Figure:**
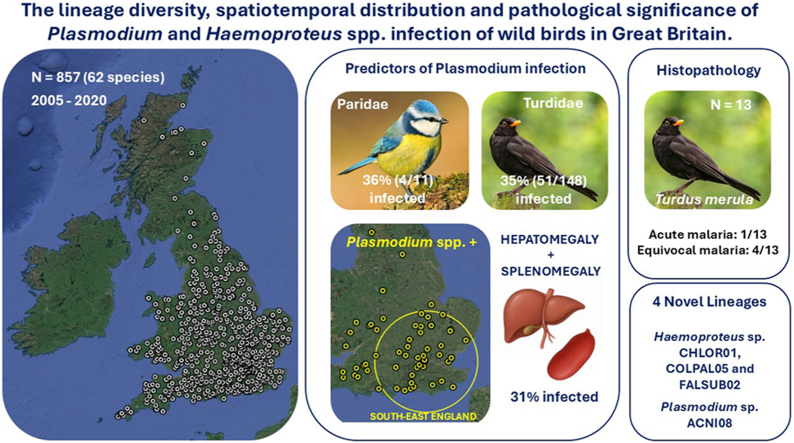

## 1 Introduction

Avian haemosporidian parasites (AHPs) form a group of cosmopolitan protists, of which *Plasmodium* and *Haemoproteus* are the two genera most frequently detected in avian hosts (Valkiūnas, 2005). Over 250 species of AHP have been described morphologically, comprising more than 4500 lineages affecting at least 2000 avian host species across six continents (Valkiūnas, 2005; MalAvi, 2021).

Avian haemosporidian parasites undergo complex, heteroxenous life cycles, which involve both asexual and sexual reproduction in avian and arthropod hosts, respectively (Atkinson et al., 2008; Valkiūnas, 2005). Parasites of the genus *Plasmodium* are transmitted by female mosquitoes (*Culicidae*). In western Europe, *Culex pipiens* appears to be an important vector for avian *Plasmodium* spp., although other mosquito species have also been implicated (Alves, 2012; Schoener et al., 2019; Ventim et al., 2012). Parasites belonging to the genus *Haemoproteus* are vectored chiefly by biting midges (*Ceratopogonidae*) and louse flies (*Hippoboscidae*), although *Haemoproteus* DNA has also been detected in mosquitoes, prompting further investigation into the competence of these potential vectors (Ferraguti et al., 2013; Gutiérrez-López et al., 2016; Santiago-Alarcon et al., 2012).

*Plasmodium* spp. are the causative agents of avian malaria, which can manifest as acute or chronic disease (Valkiūnas, 2005). Clinical signs associated with acute avian malaria include anaemia, pyrexia and high levels of mortality in susceptible hosts (Atkinson et al., 2008). Diverse fitness costs have been reported across myriad avian taxa as a result of chronic avian malaria (Kilpatrick et al., 2006; van Riper et al., 1986). *Haemoproteus* infection, meanwhile, can lead to a range of host effects, including reduced survival and reproduction rates and mortality in some cases (Cannell et al., 2013; Ortiz-Catedral et al., 2019; Puente et al., 2010). Grossly, hepatosplenomegaly, caused by increased cellularity and phagocytic macrophage activity, is the hallmark of avian haemosporidiosis in susceptible hosts (Atkinson et al., 2008; Terio, 2019; Villegas Narvaez and Brash, 2013). Histologically, hepatic and splenic necrosis are common findings, as well as deposition of the malaria pigment hemozoin in the liver and spleen, which accumulates in host macrophages following removal of parasitised host cells (Villegas Narvaez and Brash, 2013). Exoerythrocytic meronts can also be visualised in endothelial cells and macrophages from various organs, typically the lung, liver or spleen (Terio, 2019; Villegas Narvaez and Brash, 2013).

At a population scale, susceptibility to AHP infection and subsequent disease depends largely on the immunological and evolutionary naïvety of the host, as well as the virulence of the AHP (Atkinson et al., 2008; Atkinson and LaPointe, 2009; Himmel et al., 2021; Valkiūnas, 2005). Avian populations which have evolved in isolation from AHPs, before being later exposed through pathogen pollution and/or vector translocation, have subsequently experienced mass mortality and catastrophic population declines (Atkinson and LaPointe, 2009; Sijbranda et al., 2016). The most striking and well-cited example of this is the co-introduction of *Plasmodium relictum* and a competent vector for the parasite, *Culex quinquefasciatus*, to the Hawaiian archipelago, which led to the extinction of over 20 species of native Hawaiian birds (Atkinson and LaPointe, 2009; van Riper et al., 1986). Similarly, disease and high rates of mortality have been reported in evolutionarily naïve captive avian populations exposed to AHPs to which they would not be exposed within their natural range (Grilo et al., 2016; Ortiz-Catedral et al., 2019).

In regions where AHPs are endemic and avian hosts have co-evolved alongside these parasites, infection has traditionally been considered benign, with any effects on host fitness typically being subclinical, or even potentially beneficial (Atkinson et al., 2008; Zylberberg et al., 2015). Whilst the subclinical effects of AHPs on host fitness and population dynamics remain poorly understood in co-adapted wild birds, both *Plasmodium* and *Haemoproteus* infections have been associated with lower hatching and fledging rates (Knowles et al., 2010; Marzal et al., 2005; Merino et al., 2000), as well as reduced overwinter survival (Dadam et al., 2019; Grabow et al., 2024).

The advent of molecular diagnostics has brought to light the diversity of AHPs infecting wild birds, leading to the cataloguing of a diverse assemblage of *Plasmodium*, *Haemoproteus* and *Leucocytozoon* (another avian haemosporidian parasite) spp. lineages over the last 20 years (Bensch et al., 2009). The majority of this research, however, has focussed on screening healthy birds caught in the field, providing limited data pertaining to the host effects of any identified lineages and potentially leading to under-reporting of lineages causing severe or fatal disease (Himmel et al., 2020; Mukhin et al., 2016; Wobeser, 2006).

Recent studies making concurrent use of molecular techniques and histopathology point to marked variation between AHP lineages with respect to pathogenicity and virulence within co-adapted avian populations (Dinhopl et al., 2015; Himmel et al., 2020, 2021). Host susceptibility also seems to vary widely with regards to both infection and disease outcomes. Species belonging to the families Turdidae and Paridae appear to be particularly susceptible to AHPs, with high rates of infection reported from across Europe in these taxa (Hatchwell et al., 2000; Himmel et al., 2020; Pigeault et al., 2018; Wood et al., 2007). Eurasian blackbird (*Turdus merula*; hereafter blackbird) populations in particular appear to experience a high prevalence of *Plasmodium* infection with some lineages associated with high exoerythrocytic parasite burdens causing disease and, in some cases, mortality (Dinhopl et al., 2015; Himmel et al., 2020).

Making use of tissue samples collected as part of a long term passive wildlife disease surveillance programme in Great Britain (GB), we aimed to 1) catalogue the lineages of *Plasmodium* and *Haemoproteus* spp. that infect wild birds; 2) investigate the phylogeny of *Plasmodium* and *Haemoproteus* spp. lineages detected in wild birds and examine taxonomic host-parasite associations; 3) identify predictors of *Plasmodium* and/or *Haemoproteus* spp. infection in wild birds comprising age, sex, species, body condition, season and year; 4) investigate the spatial distribution of *Plasmodium* and/or *Haemoproteus* spp. infection in wild birds; 5) appraise histological evidence of host response and disease in infected birds and 6) investigate relationships between *Plasmodium* and/or *Haemoproteus* spp. infection and post-mortem examination (PME) findings.

## 2 Materials and methods

### 2.1 Sample collection

This study made use of a convenience sample collected during the period 2005–2020 inclusive, through a national passive wildlife disease surveillance programme in GB (the Garden Bird Health initiative; 2005–2012, and subsequently the Garden Wildlife Health project, www.gardenwildlifehealth.org; 2013–2020). During this period, wild bird carcasses were submitted by members of the public and underwent PME according to a standardised protocol (Lawson et al., 2010). The majority of carcasses submitted were found in peridomestic habitats, such as gardens. Data relating to case history, morphometrics, age, sex, gross pathological, parasitological and microbiological findings were recorded for each bird examined. Tissue samples were frozen at −80 °C for later analysis and collected in neutral-buffered 10 % formalin when the state of carcass preservation was considered adequate to permit meaningful histological examination. Where possible, a cause of death was assigned based on PME findings and ancillary diagnostics (i.e. gross examination, microbiology, parasitology).

A total of 3720 birds were examined post-mortem over the period April 2005–December 2020 inclusive. Of these, tissues from 857 individual birds of eight orders, 27 families and 62 species were screened for the presence of *Plasmodium* and *Haemoproteus* by PCR.

In an effort to achieve broad taxonomic coverage, reflecting the composition of the available frozen sample archive, a sub-sample, comprising at least 10 % of cases of each taxonomic family examined, was screened by PCR. For ‘priority families’ (i.e. families previously reported to exhibit high prevalence of, or susceptibility to, AHP infection, namely Turdidae and Passeridae), at least 25 % of cases were screened. Cases were selected based on the availability of liver and spleen tissue and to ensure a wide spatial (i.e. from 648 sites from across Great Britain) and temporal (i.e. variation by year and month over the study period) distribution.

### 2.2 DNA extraction

DNA was extracted from pooled liver and spleen, or liver alone, using the Qiagen DNeasy Blood and Tissue Kit (Qiagen) according to the manufacturer's protocol for tissue samples. For the majority of cases, both liver and spleen tissues were available, with 12.5 mg of each tissue used for DNA extraction. In cases for which spleen was unavailable, 25 mg of liver was used. Negative extraction controls, consisting of 25 μl of sterile nuclease-free water, were included and all DNA extracts were stored at −20 °C prior to PCR.

### 2.3 Molecular analyses

DNA extracts were screened for *Plasmodium* and *Haemoproteus* using the nested PCR protocol described by Waldenström et al. (2004), targeting the cytochrome *b* gene. The initial round of PCR contained primers HaemNF (5′– CATATATTAAGAGAATTATGGAG –3′) and HaemNR2 (5′– AGAGGTGTAGCATATCTATCTAC –3′). Each first round reaction had a total volume of 13 μl and contained 6.25 μl HotStar Taq Master Mix (Qiagen, UK), 1 μl of each primer (10 pmol/μl), 2.75 μl RNase-free water and 2 μl extracted DNA. The thermal profile comprised a 15-min activation step at 95 °C, followed by 25 cycles of 94 °C for 1 min, 50 °C for 1 min and 72 °C for 1 min, finishing with an elongation step at 72 °C for 10 min. The second, nested PCR reaction, included the primers HaemF (5′– ATGGTGCTTTCGATATATGCATG –3′) and HaemR2 (5′–GCATTATCTGGATGTGATAATGGT –3′). Each reaction contained a total volume of 25 μl with 12.5 μl HotStar Taq Master Mix (Qiagen, UK), 4 μl of each primer (10 pmol/μl), 2.5 μl of RNase-free water and 2 μl of the first run PCR product. The thermal profile was identical to the first reaction, but with an increased number of cycles from 25 to 35. Positive and negative PCR controls were included in each reaction plate, comprising the same reagents with template DNA from a known *Plasmodium*-positive sample and 5 μl of sterile nuclease-free water, respectively.

Products from all reactions were run on 1–2 % agarose gel and amplicons of an anticipated length (∼500bp) were purified using either a QIAquick Gel Extraction kit or QIAquick PCR Purification Kit (Qiagen, UK) according to the manufacturer's protocols and submitted to a commercial laboratory for bi-directional Sanger sequencing. Sequence data were visually inspected and assembled using Chromas 2.6.6 (Technelysium Ltd), before being subjected to BLAST search in both the National Centre for Biotechnology Information's (NCBI) GenBank and the MalAvi database (Bensch et al., 2009). A sample was only considered positive for *Haemoproteus* or *Plasmodium* following assembly of a high-quality, unambiguous bi-directional consensus sequence. Sequences were considered homologous with published lineages only when a 100 % match was found. Sequences which were not identical to any published lineages were assigned new lineage names according to MalAvi guidelines and deposited in the GenBank database (Accession numbers PV849993-PV849996; Bensch et al., 2009).

### 2.4 Phylogenetic analyses

Following sequence assembly using Chromas 2.6.6 (Technelysium Ltd), maximum likelihood analysis was performed using MEGA11 (www.megasoftware.net) according to the Tamura-Nei model (Tamura and Nei, 1993). Support for internal branches underwent bootstrap analysis with 1000 replications. Two species of *Plasmodium* known to infect mammalian hosts (*P. falciparum* [AJ298787] and *P. reichenowil* [AF069610]) were selected as an outgroup to root the phylogenetic tree.

### 2.5 Histopathology

Histological examinations were focused on blackbirds since this species is known to experience high rates of *Plasmodium* infection, sometimes associated with significant disease, in mainland Europe (Dinhopl et al., 2015; Himmel et al., 2020). All PCR-positive blackbirds with available fixed tissues were allocated a preservation score of 1–6 depending on the degree of autolysis and whether or not the carcass had been frozen prior to PME. A subset of tissue samples from PCR-positive blackbirds with a preservation score of ≤2 (i.e. no or mild autolysis and not frozen prior to fixation) were selected and processed for histological examination using routine methods. Sections of a range of tissues, including liver, lung and spleen, were stained as routine with haematoxylin and eosin, and special stains comprising Gram, Perls’ Prussian blue, periodic acid Schiff and Ziehl-Neelsen were used as indicated. Sections were independently examined by two veterinary pathologists (authors AAC and SS), with consensus results reported.

Histological findings were recorded according to a standardised protocol, with scores of 0–3 allocated, based on the degree of autolysis (0 = no autolysis; 1 = mild autolysis; 2 = moderate autolysis; 3 = advanced autolysis); presence of malarial lesions; presence of the malarial pigment, hemozoin; presence of exoerythrocytic meronts and degree of haemosiderosis observed. Other significant findings (e.g. comorbidities) were also recorded.

### 2.6 Statistical analyses

Results from PCR screening were analysed using univariate binary logistic regression in R Studio (R Core Team, 2021) to identify any significant predictor variables for the presence of *Haemoproteus* or *Plasmodium* infection. Predictor variables assessed comprised taxonomic order, family and species, sex, age class (‘juvenile’ refers to nestling and fledgling birds sampled prior to completing post-juvenile moult and ‘adult’ refers to all birds which had completed post-juvenile moult at the time of sampling), organomegaly (i.e. presence of hepatomegaly and/or splenomegaly), body condition, month and year of carcass discovery and cause of death category (e.g. undetermined, trauma, infectious disease, predation). Spatial clustering of PCR-positive birds was investigated using SatScan spatial analysis software, assessing the distribution of infected birds against a background population of all birds screened by PCR (www.satscan.org).

## 3 Results

### 3.1 Molecular and phylogenetic analyses

The overall infection rate with *Plasmodium* and/or *Haemoproteus* was 13.5 % (116/857) but this varied markedly between families (Table 1). *Plasmodium* and *Haemoproteus* infection rates were 8.9 % (76/857) and 4.7 % (40/857), respectively, with no evidence of co-infection with multiple AHPs. *Plasmodium* infection was detected in birds belonging to the orders Passeriformes (11.4 %; 73/640), Strigiformes (9.1 %; 1/11) and Accipitriformes (4.8 %; 2/42). Infection with *Haemoproteus* was detected in Columbiformes (10.6 %; 14/132), Strigiformes (9.1 %; 1/11), Falconiformes (8.3 %; 1/12) and Passeriformes (3.8 %; 24/640).

**Table 1:** *Plasmodium* and *Haemoproteus* PCR screening results according to host taxonomic family. Total number and percentages provided. AHP = avian haemosporidian parasite.

Family	Total screened	Number *Haemoproteus*-positive (%)	Number *Plasmodium*-positive (%)	Number AHP-positive (%)
Accipitridae	42	–	2 (4.8 %)	2 (4.8 %)
Aegithalidae	6	–	–	–
Apodidae	1	–	–	–
Bombycillidae	8	–	–	–
Certhidae	1	–	–	–
Columbidae	132	14 (10.6 %)	–	14 (10.6 %)
Corvidae	15	1 (6.7 %)	2 (13.3 %)	3 (20.0 %)
Emberizidae	9	–	–	–
Falconidae	12	1 (8.3 %)	–	1 (8.3 %)
Fringillidae	247	14 (5.7 %)	7 (2.8 %)	21 (8.5 %)
Hirundinidae	11	–	–	–
Motacillidae	1	–	–	–
Muscicapidae	34	–	3 (8.8 %)	3 (8.8 %)
Paridae	11	–	4 (36.4 %)	4 (36.4 %)
Passeridae	64	–	2 (3.1 %)	2 (3.1 %)
Phylloscopidae	1	–	–	–
Picidae	14	–	–	–
Prunellidae	34	–	3 (8.8 %)	3 (8.8 %)
Psittacidae	5	–	–	–
Regulidae	6	–	–	–
Sittidae	4	1 (25.0 %)	–	1 (25.0 %)
Strigidae	8	1 (12.5 %)	1 (12.5 %)	2 (25.0 %)
Sturnidae	26	–	1 (3.8 %)	1 (3.8 %)
Sylviidae	10	1 (3.8 %)	–	1 (3.8 %)
Troglodytidae	4	–	–	–
Turdidae	148	7 (4.7 %)	51 (34.5 %)	58 (39.2 %)
Tytonidae	3	–	–	–
**Total**	**857**	**40 (4.7 %)**	**76 (8.9 %)**	**116 (13.5 %)**

**Fig. 1:**
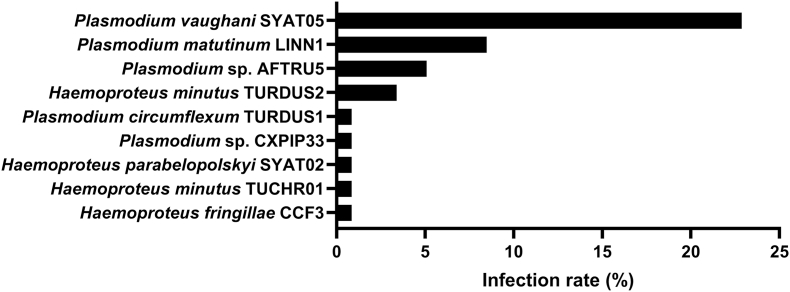
Avian haemosporidian parasite lineages detected in Eurasian blackbird (*Turdus merula*) in Great Britain. Percentages describe infection rate for the stated lineage in each species shown.

**Fig. 2:**
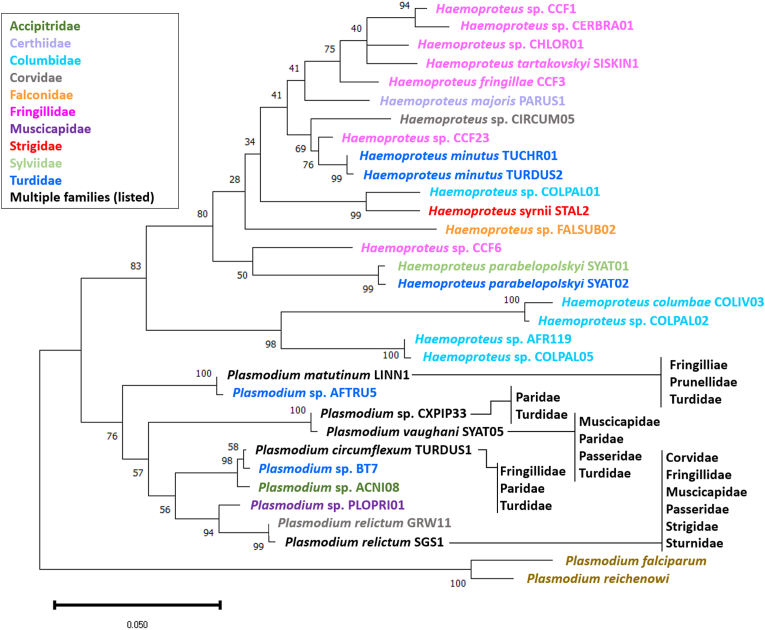
Molecular phylogenetic analysis of avian *Plasmodium* and *Haemoproteus* spp. detected in wild birds in Great Britain, constructed by Maximum Likelihood method based on trimmed 480 bp cytochrome B sequences, with 1000 bootstrap repetitions. The numbers next to branches indicate the percentage of trees in which the associated taxa clustered together. The tree is drawn to scale with branch lengths representing the number of substitutions per site. Evolutionary analyses were conducted in MEGA12. Lineage name colour indicates the host family in which the lineage has been detected in Great Britain. For lineages detected in multiple host families, the host families are listed.

Sequence analysis of PCR products, which ranged from 340 bp to 523 bp in length, revealed a total of 30 AHP lineages, 20 belonging to the genus *Haemoproteus* and 10 to the genus *Plasmodium* (Table 2). Twenty-four of these were not previously recorded in GB, of which two represent first detections in Europe and four are apparently novel lineages. Fig. 1 describes the *Plasmodium* and *Haemoproteus* lineages infecting birds of the families Paridae and Turdidae, which exhibited the highest rates of both *Plasmodium* infection (36.4 % and 34.5 %, respectively) and AHP infection overall (36.4 % and 39.2 %, respectively). A maximum-likelihood phylogenetic tree was constructed based on a 480 bp section of the cytochrome B gene (Fig. 2), selected on the basis that this was the maximum length from which at least one example of each of the *Plasmodium* and *Haemoproteus* lineages detected in this study was available.

**Table 2:** *Plasmodium* and *Haemoproteus* lineages detected in wild birds in Great Britain. Lineage names in bold type indicate novel lineages. Lineages marked with an asterisk indicate those which have not been previously reported in Great Britain.

Parasite species	Parasite lineage	Host family	Host species (number and percentage infected)
*Haemoproteus columbae*	COLIV03 ∗	Columbidae	*Columba livia* (1/24; 4.2 %)
*Haemoproteus fringillae*	CCF3 ∗	Fringillidae Turdidae	*Fringilla coelebs* (1/60; 1.7 %) *Spinus spinus* (1/26; 3.8 %) *Turdus merula* (1/118; 0.8 %)
*Haemoproteus* sp.	**CHLOR01** ∗	Fringillidae	*Chloris chloris* (1/95; 1.1 %)
*Haemoproteus majoris*	PARUS1 ∗	Sittidae	*Sitta europaea* (1/4; 25 %)
*Haemoproteus minutus*	TUCHR01 ∗	Turdidae	*Turdus merula* (1/118; 0.8 %)
*Haemoproteus minutus*	TURDUS2	Turdidae	*Turdus merula* (4/118; 3.4 %)
*Haemoproteus parabelopolskyi*	SYAT01 ∗	Sylviidae	*Sylvia atricapilla* (1/7; 14.3 %)
*Haemoproteus parabelopolskyi*	SYAT02 ∗	Turdidae	*Turdus merula* (1/118; 0.8 %)
*Haemoproteus* sp.	AFR119 ∗	Columbidae	*Columba palumbus* (7/38; 18.4 %) *Columba livia* (1/24; 4.2 %)
*Haemoproteus* sp.	CCF23 ∗	Fringillidae	*Fringilla coelebs* (1/60; 1.7 %)
*Haemoproteus* sp.	CCF6 ∗	Fringillidae	*Fringilla coelebs* (4/60; 6.7 %) *Chloris chloris* (1/95; 1.1 %)
*Haemoproteus* sp.	CERBRA01 ∗	Fringillidae	*Fringilla coelebs* (2/60; 3.3 %)
*Haemoproteus* sp.	CIRCUM05 ∗	Corvidae	*Garrulus glandarius* (1/1; 100.0 %)
*Haemoproteus* sp.	COLPAL01 ∗	Columbidae	*Columba palumbus* (1/38; 2.6 %)
*Haemoproteus* sp.	COLPAL02 ∗	Columbidae	*Columba palumbus* (1/38; 2.6 %) *Streptopelia decaocto* (1/60; 1.7 %)
*Haemoproteus* sp.	**COLPAL05** ∗	Columbidae	*Columba palumbus* (2/38; 5.3 %)
*Haemoproteus* sp.	**FALSUB02** ∗	Falconidae	*Falco subbuteo* (1/1; 100.0 %)
*Haemoproteus syrnii*	STAL2 ∗	Strigidae	*Strix aluco* (1/7; 14.3 %)
*Haemoproteus tartakovskyi*	SISKIN1 ∗	Fringillidae	*Spinus spinus* (1/26; 3.8 %)
*Hemoproteus* sp.	CCF1 ∗	Fringillidae	*Chloris chloris* (1/95; 1.1 %) *Fringilla coelebs* (1/60; 1.7 %)
*Plasmodium circumflexum*	TURDUS1	Fringillidae Paridae Turdidae	*Fringilla coelebs* (3/60; 5.0 %) *Periparus ater* (2/10; 20.0 %) *Buteo buteo* (1/28; 3.6 %) *Turdus merula* (1/118; 0.8 %)
*Plasmodium matutinum*	LINN1	Fringillidae Prunellidae Turdidae	*Turdus merula* (10/118; 8.5 %) *Turdus philomelos* (2/19; 10.5 %) *Chloris chloris* (1/95; 1.1 %) *Prunella modularis* (1/34; 2.9 %)
*Plasmodium relictum*	GRW11	Corvidae	*Pica pica* (1/30; 33.3 %)
*Plasmodium relictum*	SGS1	Corvidae Fringillidae Muscicapidae Passeridae Strigidae Sturnidae	*Fringilla coelebs* (2/60; 3.3 %) *Athene noctua* (1/1; 100.0 %) *Carduelis carduelis* (1/28; 3.6 %) *Erithacus rubecula* (1/30; 3.3 %) *Passer montanus* (1/10; 10.0 %) *Pica pica* (1/3; 33.3 %) *Prunella modularis* (1/34; 2.9 %) *Sturnus vulgaris* (1/26; 3.8 %)
*Plasmodium* sp.	**ACNI08** ∗	Accipitridae	*Accipiter nisus* (1/13; 7.7 %)
*Plasmodium* sp.	AFTRU5 ∗	Turdidae	*Turdus merula* (6/118; 5.1 %) *Turdus philomelos* (1/19; 5.3 %)
*Plasmodium* sp.	BT7	Turdidae	*Turdus pilaris* (1/3; 33.3 %)
*Plasmodium* sp.	CXPIP33 ∗	Turdidae Paridae	*Turdus merula (1/118; 0.8 %)* *Poecile palustris (1/1; 100 %)*
*Plasmodium* sp.	PLOPRI01 ∗	Muscicapidae	*Muscicapa striata* (1/3; 33.3 %)
*Plasmodium vaughani*	SYAT05	Muscicapidae Paridae Passeridae Turdidae	*Turdus merula* (27/118; 22.9 %) *Turdus philomelos* (2/9; 10.5 %) *Erithacus rubecula* (1/30; 3.3 %) *Passer domesticus* (1/54; 1.9 %) *Periparus ater* (1/10; 10.0 %) *Prunella modularis* (1/34; 2.9 %)

### 3.2 Spatial analyses

**Fig. 3:**
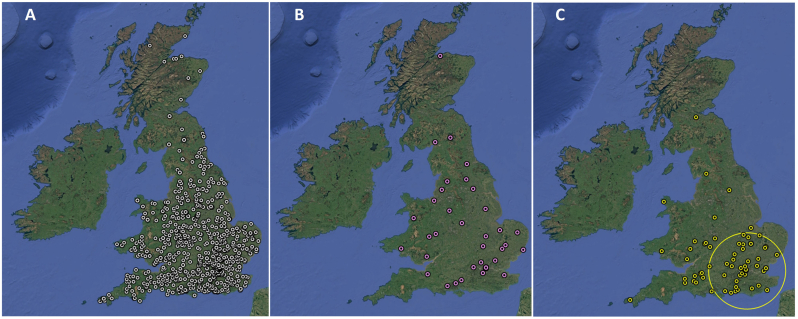
A: Spatial distribution of all wild birds screened for presence of avian haemosporidian parasites of the genera *Plasmodium* and *Haemoproteus* in Great Britain. B: Spatial distribution of all cases testing PCR-positive for *Haemoproteus* infection. C: Spatial distribution of all cases testing PCR-positive for *Plasmodium* sp. infection. Yellow circle shows spatial cluster of *Plasmodium* spp. infection (diameter = 128.4 km; centred on point 51.489838 N, 0.099212 W). Map was created with Google Earth Pro. Version 7.March 3, 7699 (2022) (https://www.google.com/intl/en_uk/earth/versions/#earth-pro).

Of the 857 birds screened for *Plasmodium* and *Haemoproteus* infection, 765 were from England, 81 from Wales and 11 from Scotland (Fig. 3A). Spatial analyses revealed a single significant spatial cluster of *Plasmodium*-positive cases in Southeast England (Fig. 3C). No significant clusters of *Haemoproteus* infection were identified (Fig. 3B).

### 3.3 Predictors of *Plasmodium* and *Haemoproteus* infection

Organomegaly at PME, cause of death category, taxonomic family, month and region of carcass discovery were all found to be statistically significant predictors of *Plasmodium* infection (Table S1), whilst host age class, sex, order, body condition and year of carcass discovery were not. No variables were found to be statistically significant predictors of *Haemoproteus* infection.

Analysis of the entire dataset revealed that identification of splenomegaly only (Odds Ratio [OR] = 3.7; p < 0.001), hepatomegaly only (OR = 3.5; p < 0.001) and hepatosplenomegaly (OR = 5.0; p < 0.001) at PME were all statistically significant predictors of infection with *Plasmodium* but not *Haemoproteus* (Fig. S1). However, when blackbirds were excluded from the analysis, none of these variables remained statistically significant predictors of *Plasmodium* infection. When considering only blackbirds, splenomegaly (OR = 4.5; p = 0.005) and hepatosplenomegaly (OR = 6.5; p = 0.02) remained statistically significant predictors of *Plasmodium* infection but hepatomegaly did not (OR = 2.7; p = 0.07).

### 3.4 Histopathology

Thirteen PCR-positive blackbirds were selected for histological examination and scored according to the degree of autolysis, presence of exoerythrocytic meronts, hemozoin deposition in tissues, haemosiderosis and the presence of other lesions consistent with avian malaria (Table S2). All 13 blackbirds exhibited some degree of autolysis, with most cases attributed a score of two (n = 5) or three (n = 5), indicating moderate or advanced autolysis, respectively. Three birds were diagnosed with concomitant infectious disease: two with Usutu viral (USUV) disease (Lawson et al., 2022) and one with systemic isosporiasis (Yaffy et al., *unpublished data*). Meronts were detected in only four birds and malarial pigment, hemozoin, in seven. Histopathological lesions consistent with clinical malaria (i.e. splenic and hepatic necrosis) were detected in five of the 13 blackbirds examined. Only one bird, which was infected with *Plasmodium vaughani* SYAT05, was diagnosed with acute malaria, whereas the remaining four with visible meronts exhibited equivocal lesions that could not be conclusively attributed to the parasites. Two of these birds were infected with *P. vaughani* SYAT05, one with *Plasmodium matutinum* LINN1 and one with *Plasmodium* sp. AFTRU5 (Table S2). The cause of death assigned at PME was undetermined in the bird diagnosed with acute malaria by histology and in three of the cases with equivocal findings. In the remaining equivocal case, the cause of death was attributed to predation.

## 4 Discussion

Through utilising citizen science to deliver wildlife disease surveillance, this study has led to the identification of 30 unique lineages of AHP in wild birds in GB, infecting 27 species, belonging to five orders and 14 families. Of the 30 lineages detected, four are apparently novel (*Haemoproteus* sp. CHLOR01 [PV849994], COLPAL05 [PV849995] and FALSUB02 [PV849996] and *Plasmodium* sp. ACNI08 [PV849993]), and only seven have previously been reported in GB (*P. matutinum* LINN1; *P. relictum* GRW11 and SGS1; *Plasmodium* sp. BT7; *P. vaughani* SYAT05; *Plasmodium circumflexum* TURDUS1 and *Plasmodium minutus* TURDUS2; Cosgrove et al., 2008; González-Olvera et al., 2022; Wood et al., 2007). Two of the detected lineages (*Haemoproteus columbae* COLIV03 and *Haemoproteus* sp. COLPAL01) have not been previously reported from Europe (Bensch et al., 2009).

Overall infection rates, 8.9 % (76/857) for *Plasmodium* and 4.7 % (40/857) for *Haemoproteus,* were slightly lower than those reported from a similar study conducted in Austria, where overall infection rates of 11 % (9/81) for *Plasmodium* and 12 % (10/81) for *Haemoproteus* were detected (Himmel et al., 2021). Due to the inherently biased nature of the convenience sampling methodologies used in both studies, neither value represents infection ‘prevalence’ in wild birds and comparisons between such studies must be made with caution. This observed discrepancy in infection rate may be explained by varying taxonomic composition of sample sets, as well as seasonal variation, with Himmel et al. (2021) only screening birds collected between the months of June and October, when infection rates would be expected to be highest due to greater vector abundance and environmental conditions which facilitate AHP sporogony (Cosgrove et al., 2008).

The families Turdidae (35 %; 51/148) and Paridae (36 %; 4/11) exhibited the highest levels of *Plasmodium* infection in the present study, which is consistent with previous reports of high AHP prevalence in these taxa (Hatchwell et al., 2000; Hellgren et al., 2011; Himmel et al., 2020). The *Plasmodium* infection rate was particularly high in blackbirds, with 38.1 % (45/118) testing PCR-positive. The only previous study of AHPs in blackbirds in GB reported a *Plasmodium* prevalence of 32.6 % (32/98); though these figures are similar, the latter study used blood smear examination and may therefore have underestimated the true prevalence due to the lower sensitivity of this method compared to molecular techniques (Bensch et al., 2000; Hatchwell et al., 2000; Waldenström et al., 2004). Studies from other countries have reported similarly high rates of infection in blackbirds, both inside and outside their native range (Ewen et al., 2012; Himmel et al., 2020, 2021; Rouffaer et al., 2018). It is possible that the prioritisation of samples from the families Paridae and Turdidae, whereby at least 25 % of archived samples from these two families were included in the study as opposed to a minimum of 10 % of all other families, may have introduced a degree of bias when comparing infection rates between families. It is important to bear in mind, however, that the study made use of a convenience sample which inherently introduces a degree of bias and birds were selected only on the basis of tissue availability and spatiotemporal spread and were not prioritised according to suspicion of AHP infection.

Passeridae exhibited a relatively low rate of *Plasmodium* infection (3 %; 2/64), contrary to previous data from GB and mainland Europe. Whereas only a single house sparrow (2 %; 1/54) was found to be infected in this study, Dadam et al. (2019) identified *Plasmodium* infection rates in populations of house sparrows (*Passer domesticus*) sampled in Greater London that averaged 74 % (n = 380), and varied between 50 and 100 % across sites. The high prevalence reported by Dadam et al. (2019) may be highly localised, and indeed the one *Plasmodium*-positive house sparrow in this study came from a site within Greater London. However, a further 15 house sparrows from Greater London and Southeast England were screened and none yielded a positive result. In France, Bichet et al. (2014) screened 16 populations of house sparrow and found *Plasmodium* prevalence ranged from 11 to 79 % across populations. Meanwhile, Minayo Martín et al. (2025) compared house sparrows occupying urban and rural habitats in central and southern Spain and found an overall prevalence of 64 %, with a higher prevalence in urban populations than their rural equivalents. The reason for the comparatively low rates of *Plasmodium* and *Haemoproteus* infection in house sparrows found in this study is unclear but may be due to varying methodologies. In this study, liver and spleen samples were analysed from birds found dead, whilst the aforementioned research screened blood samples taken from live birds by PCR. Also, Himmel et al. (2021) found no evidence of AHP infection in the small number of *Passer* spp. found dead in Austria and screened using similar methods to this study.

Of the 27 lineages detected, *P. vaughani* SYAT05 was the most prevalent, having been detected in 35 birds of six species and five families. *Plasmodium vaughani* SYAT05 is amongst the most widely reported AHP lineages and infection has been reported in 31 avian species from every continent except Antarctica (Drovetski et al., 2014; Ewen et al., 2012; Martinsen et al., 2007; Mata et al., 2015). Although exceptions exist, *Haemoproteus* parasites tend to exhibit a greater degree of host specificity compared to *Plasmodium* parasites (Fecchio et al., 2020; Olsson-Pons et al., 2015; Valkiūnas, 2005). Consistent with this, three quarters (15/20) of the *Haemoproteus* lineages detected here were found in only a single host species, compared to just half (5/10) of *Plasmodium* lineages. All but one of the *Haemoproteus* lineages were found in hosts of a single family, with the remaining lineage affecting birds in two families. By comparison, only five of ten of the identified *Plasmodium* lineages were limited to a single host family and *P. relictum* SGS1 was identified in six families across two orders. Phylogenetic analysis of *Haemoproteus* lineages revealed clear host-specificity within phylogenetic clusters, in contrast to the apparently more generalist *Plasmodium* lineages. For example, the *Haemoproteus* lineages AFR119, COLIV03 and COLPAL02 clustered closely within the phylogenetic tree and were all identified exclusively in the family *Columbidae*, consistent with reports from mainland Europe and Africa (Mata et al., 2015; Nebel et al., 2020; Schumm et al., 2021).

Hepatosplenomegaly, as well as either hepatomegaly or splenomegaly alone, were found to be significant predictors of *Plasmodium* infection across the sample set. Whilst it is possible that the organomegaly observed was caused by *Plasmodium* and/or *Haemoproteus* infection, this cannot be confirmed in the absence of histopathological examination of liver and spleen from all cases. Indeed, of the 13 blackbirds examined histologically, though eight exhibited some degree of either hepatomegaly or splenomegaly at PME, only one was definitively diagnosed with acute malaria as the cause. Additionally, other infectious conditions, both diagnosed and undiagnosed, may confound these results. For example, USUV disease or systemic isosporiasis, both of which were diagnosed in birds included in these analyses, may also be associated with hepatic or splenic enlargement.

It is also noteworthy that organomegaly was no longer a useful predictor of *Plasmodium* infection when blackbirds were excluded from the analyses. However, it remains unknown whether this may be due to host factors associated with blackbirds (or other species), such as immunocompetence, or perhaps relates to pathogen factors, such as the pathogenicity of the lineage/s most-frequently infecting different wild bird species. As mentioned previously, *Plasmodium* infection appears to be particularly prevalent in blackbirds in Europe: indeed this species exhibited the highest infection rate of any species in this study (38.1 %; n = 118). Himmel et al. (2020) reported similarly high rates of *Plasmodium* infection in blackbirds submitted as part of a wildlife disease surveillance scheme in Austria (64 %; n = 277) and AHP infection rates exceeding 80 % have been reported in blackbirds from other sites in mainland Europe (Bentz et al., 2006; Hatchwell et al., 2000; Rouffaer et al., 2018). A future line of enquiry could therefore be to investigate whether blackbirds may be comparatively more likely to develop organomegaly in association with either clinical or sub-clinical AHP infection, relative to other species.

Birds for which no putative cause of death could be identified based on PME findings were more likely to be infected with *Plasmodium* than those for which a cause of death was determined. One possible explanation for this trend is the inability to diagnose acute malaria without histopathological examination and that a proportion of birds for which no cause of death could be identified may have died as a result of undiagnosed acute malaria. Three of the four equivocal cases of possible malaria were assigned an undetermined cause of death based on PME prior to molecular diagnostics, as was the single bird in which acute malaria was unequivocally diagnosed. Whilst it is possible that malaria contributed to the death of these birds, in the absence of a definitive diagnosis of malaria in all but one of these cases, inferences which can be made are limited.

Histological examination of tissues from thirteen *Plasmodium*-infected blackbirds revealed unequivocal evidence of acute malaria in just one case: a juvenile male infected with *P*. *vaughani* SYAT05 which exhibited severe, focally extensive, necrotising heterophilic hepatitis and splenitis. Unfortunately, histological examination of more specimens was prohibited by the degree of autolysis affecting most samples. This is an intrinsic shortcoming of passive, citizen-science based wildlife disease surveillance schemes as the time from carcass discovery to examination often measures in days rather than hours, during which time autolysis occurs rapidly. Chromogenic *in situ* hybridization (CISH), visualising target DNA sequences within tissue sections or cells, may offer a potential solution to this problem and shows some promise in identifying exoerythrocytic meronts in less well-preserved avian samples (Dinhopl et al., 2015). Whilst beyond the scope of this study, further work conducting histopathological examinations on a wider range of wild bird species is merited to broaden our understanding of the significance of AHP infection to host health across taxa.

We found no evidence of disease associated with *P. matutinum* LINN1 infection in blackbirds, in contrast to findings in mainland European populations (Dinhopl et al., 2015; Himmel et al., 2020; Rouffaer et al., 2018). Himmel et al. (2020) reported high exo-erythrocytic parasite burdens in blackbirds infected with this lineage and noted histopathological lesions in LINN1-infected birds consistent with acute malaria, with around 20 % of LINN1-infected blackbirds exhibiting high exoerythrocytic parasite burdens in brain and/or lung tissue. Here, only one of the four LINN1-infected blackbirds examined histologically showed equivocal lesions potentially consistent with acute malaria. The only blackbird in which acute malaria was definitively diagnosed on histopathological examination was infected with *P. vaughani* SYAT05, a lineage which was found to be common and occasionally associated with heavy exoerythrocytic parasite burdens in blackbirds in Austria (Himmel et al., 2020). Unfortunately, the methodology used here prohibited quantification of the intensity of *Plasmodium* or *Haemoproteus* infection in a given sample, increasing reliance on histopathology to ascertain the pathological significance of a given infection. Where standard PCR gives a binary positive or negative result depending on the presence or absence of AHP DNA, the use of quantitative PCR in future studies could help to gauge the intensity of infection, even in cases in which tissue preservation prohibits histopathological examination.

The spatial cluster of *Plasmodium* infection in Southeast England may be explained by geographic variation in vector abundance, by climatic conditions shortening the extrinsic incubation period and enabling a greater degree of parasite transmission, or by a combination of these factors. *Plasmodium* spp. are vectored exclusively by mosquitoes, with *C. pipiens* thought to be an important vector in western Europe and shown to be a competent vector of *P. vaughani* SYAT05 (Alves, 2012; Schoener et al., 2019; Ventim et al., 2012), the dominant lineage detected in this study. A comprehensive survey of *C. pipiens* conducted across England and Wales in 2023 showed that, whilst the mosquito was widespread, the probability of encountering *C. pipiens* was highest in the South of England and comparatively low in the north and west of the country (Widlake et al., 2025). This pattern of heterogeneous vector abundance is consistent with other countries in northern Europe (Hesson et al., 2011). Using ensemble bioclimatic modelling across a pan-European dataset, Marcolin et al. (2025) identified a strong positive correlation between *C. pipiens* abundance and human-modified, low-elevation areas, which may be driving increased *Plasmodium* and *Haemoproteus* transmission in Southeast England. Alternatively, or concomitantly, the clustering may be explained by climatic effects that could drive more rapid sporogony and replication of the parasite in Southeast England, which experiences higher mean temperatures than other regions of GB (Met Office, 2021), potentially driving more rapid sporogony and replication of the parasite. Whilst this has not been investigated in temperate systems, in Hawaii, temperatures of <13 °C were found to impair sporogony of *Plasmodium* parasites inside the vector *Culex quinquefasciatus* (LaPointe et al., 2010).

The highest *Plasmodium* and/or *Haemoproteus* infection rate was recorded for birds submitted in the month of August (27.6 %; 37/134), which, alongside April, was found to be a significant predictor of infection. The lowest infection rates were detected in birds submitted in December (0 %; 0/24). This finding is consistent with seasonality observed in other studies (e.g. Cosgrove et al., 2008; Neto et al., 2020), with possible explanations including seasonal variations in vector abundance and a shift in host population composition to a larger proportion of immunologically naïve first-year birds in the spring and summer (Cosgrove et al., 2008). Juvenile birds were not found to have an increased likelihood of infection in this study; however, a sampling bias towards adult birds, which comprised over three quarters of the birds tested, must be acknowledged.

Coinfection with multiple AHPs was not detected in any birds in this study, with all electropherograms providing unambiguous sequence data with no double peaks to indicate sequence data from multiple AHP lineages. In the literature, occurrence of coinfections is highly variable between studies, with some reporting over 20 % of AHP infections involving multiple lineages (Himmel et al., 2020; Yusupova et al., 2023) and others detecting no AHP coinfection despite large sample sets and diverse lineage identification (Dinhopl et al., 2015). Whilst it is possible that no cases of coinfection were included in our sample set, it is more likely that some samples did in fact harbour multiple AHPs, but coinfection went undetected due to the molecular methods employed. The selectivity of the PCR protocol used in this study, which does not effectively amplify DNA from *Leucocytozoon* spp., may have contributed to the lack of coinfections detected here, particularly as Himmel et al. (2020) found that approximately 90 % of AHP coinfections in Austrian *Turdidae* involved at least one lineage of *Leucocytozoon*. The Waldenström et al., 2004a, Waldenström et al., 2004b PCR protocol was originally selected due to a primary focus on *Plasmodium* sp. and the lower number of reactions required per sample, thereby reducing financial and time costs associated with the relatively large sample size. Future work using alternative PCR protocols which also identify haemosporidia belonging to the genus *Leucocytozoon*, such as that described by Hellgren et al. (2004), would further our knowledge of AHP diversity and the importance of coinfection in wild birds in GB.

Whilst no AHP coinfections were identified, two individuals were concurrently infected with USUV and *Plasmodium* spp. (one with *P. matutinum* LINN1 and the second with *P. vaughani* SYAT05). Both were juvenile blackbirds found at a single site in Greater London during the summer of 2020 (Folly et al., 2020). Histopathological examination revealed no evidence of avian malaria in either bird, so the role that *Plasmodium* played in their deaths remains unclear. However, in light of previous reports of high rates of co-infection with these two mosquito-borne pathogens, further surveillance for both pathogens in parallel is warranted (Rijks et al., 2016; Rouffaer et al., 2018).

## 5 Conclusions

This study has furthered our understanding of the diversity of *Plasmodium* and *Haemoproteus* circulating in wild birds in GB; identified potentially useful predictors of *Plasmodium* infection in wild birds including host taxa and the presence of organomegaly; detected a spatial cluster of avian *Plasmodium* infection in Southeast England; and has provided further insight into the pathogenicity of *Plasmodium* in co-adapted avian hosts. Further work should focus on investigating the pathogenicity of a wider range of host-AHP interactions, including *Leucocytozoon*, which may be aided by the use of techniques such as CISH, as well as the role AHPs play in hosts suffering from co-infection with other vector-borne pathogens such as flaviviruses.

## CRediT authorship contribution statement

**Joseph P. Heaver:** Writing – review & editing, Writing – original draft, Project administration, Methodology, Investigation, Formal analysis, Data curation, Conceptualization. **Shinto K. John:** Writing – review & editing, Investigation. **Katharina Seilern-Macpherson:** Writing – review & editing, Methodology, Conceptualization. **Simon Spiro:** Writing – review & editing, Visualization, Software, Methodology, Investigation, Data curation. **Vicky Wilkinson:** Writing – review & editing, Supervision, Methodology, Investigation, Data curation, Conceptualization. **Andrew A. Cunningham:** Writing – review & editing, Visualization, Validation, Methodology, Investigation, Funding acquisition, Conceptualization. **Becki Lawson:** Writing – review & editing, Supervision, Methodology, Investigation, Funding acquisition, Data curation, Conceptualization.

## Funding sources

Financial support for wild bird disease surveillance over the period 2005–2012 (Garden Bird Health *initiative*) came from the following organisations: Birdcare Standards Association, British Veterinary Association, Animal Welfare Foundation, CJ Wildbird Foods, Cranswick Pet Products, Gardman Ltd and the Universities Federation for Animal Welfare. Financial support for wild bird disease surveillance over the period 2013–2020 (Garden Wildlife Health project www.gardenwildlifehealth.org) came from the Department for Environment, Food and Rural Affairs (Defra), the Welsh Government and the Animal and Plant Health Agency (APHA) Diseases of Wildlife Scheme (DoWS); and from the Banister Charitable Trust, Esmée Fairbairn Foundation, Garfield Weston Foundation and the Universities Federation for Animal Welfare. Becki Lawson and Andrew Cunningham receive financial support from Research England.

## Conflicts of interest

Neither I nor any of my co-authors have any relevant conflicts of interest to declare in relation to the submission of the manuscript “Haemosporidian parasitism of wild birds in Great Britain.”
