# Supplementary material for: The lineage diversity, spatiotemporal distribution and pathological significance of Plasmodium and Haemoproteus spp. infection of wild birds in Great Britain
Source: Int J Parasitol Parasites Wildl. 2025 Oct 18;28:101148. doi: 10.1016/j.ijppaw.2025.101148 (PMC12666445; doi:10.1016/j.ijppaw.2025.101148)
Supplement: Multimedia component 1 [file mmc1.docx]

**Supplementary Materials**

**Table S1.** Statistically significant (p≤0.05) predictors of *Plasmodium* infection in wild birds in Great Britain. *Plasmodium* infection rate refers to the percentage of birds in each subset testing PCR-positive for *Plasmodium*, with total number positive and numbers tested in brackets. 95% confidence interval provided for odds ratio values.

| **Predictor Variable** | **Plasmodium infection rate** | **Odds ratio** | **95% CI** | **p-value** |
| --- | --- | --- | --- | --- |
| **Gross organomegaly** | | | | |
| Splenomegaly | 24% (14/59) | 3.7 | 1.9 – 7.2 | <0.001 |
| Hepatomegaly | 23% (13/56) | 3.5 | 1.8 – 6.9 | <0.001 |
| Hepatosplenomegaly | 31% (8/26) | 5.0 | 2.1 – 11.9 | <0.001 |
| **Cause of death category** | | | | |
| Undetermined | 18% (23/131) | 7.9 | 1.0 – 60.8 | 0.05 |
| **Family** | | | | |
| *Turdidae* | 34% (51/148) | 10.5 | 2.4 – 45.3 | 0.002 |
| *Paridae* | 36% (4/11) | 11.4 | 1.7 – 74.7 | 0.01 |
| **Month** | | | | |
| April | 16% (9/56) | 5.4 | 1.1 – 26.0 | 0.04 |
| August | 18% (13/54) | 6.1 | 1.4 – 26.8 | 0.02 |
| **Region of UK** | | | | |
| Greater London | 15% (10/68) | 5.8 | 1.2 – 27.4 | 0.03 |
| East of England | 12% (21/170) | 4.8 | 1.1 – 21.0 | 0.04 |
| South East of England | 12% (18/133) | 4.4 | 1.0 – 19.5 | 0.05 |

**Figure S1.** *Plasmodium* infection rate in wild birds in Great Britain according to post-mortem examination findings relating to hepatomegaly and/or splenomegaly. A: Blackbird (*Turdus merula; n = 118*) only; B: All other species excluding blackbirds (n = 739).
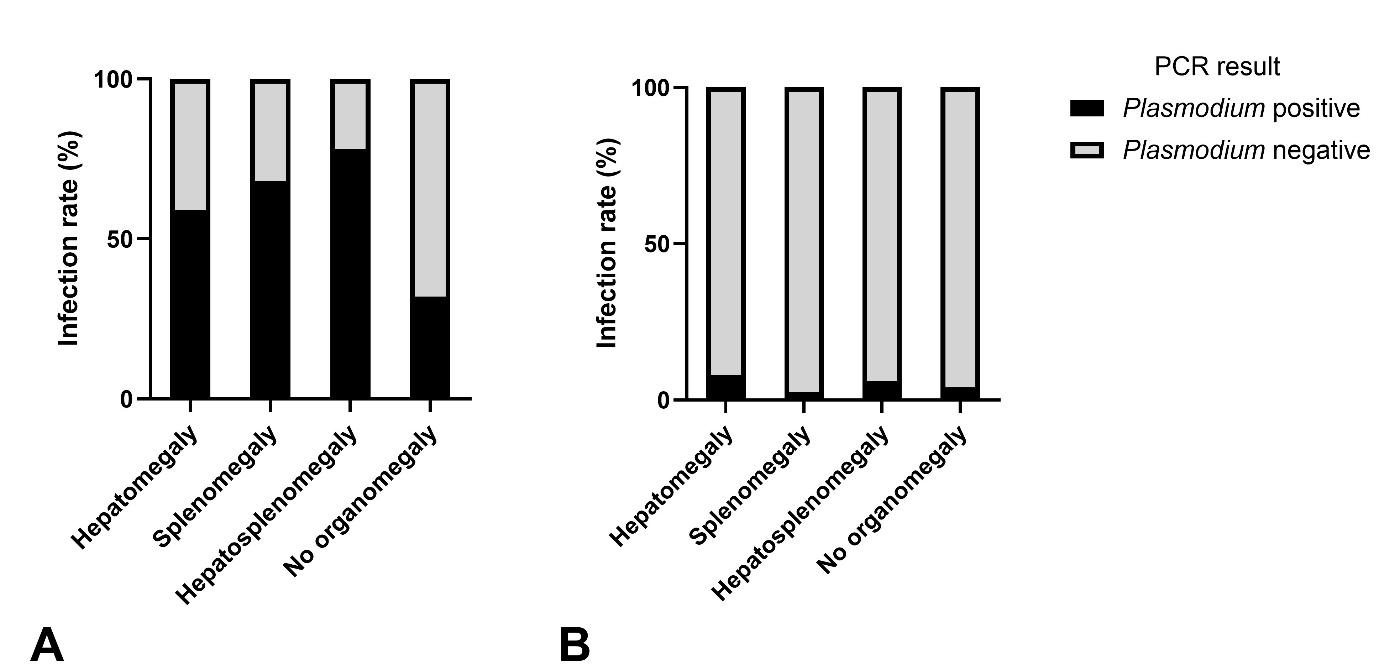


6

2

**Figure S2** Infection rate of wild birds in Great Britain with avian haemosporidian parasites of the genera *Plasmodium* and *Haemoproteus* according to month of carcass discovery.

**
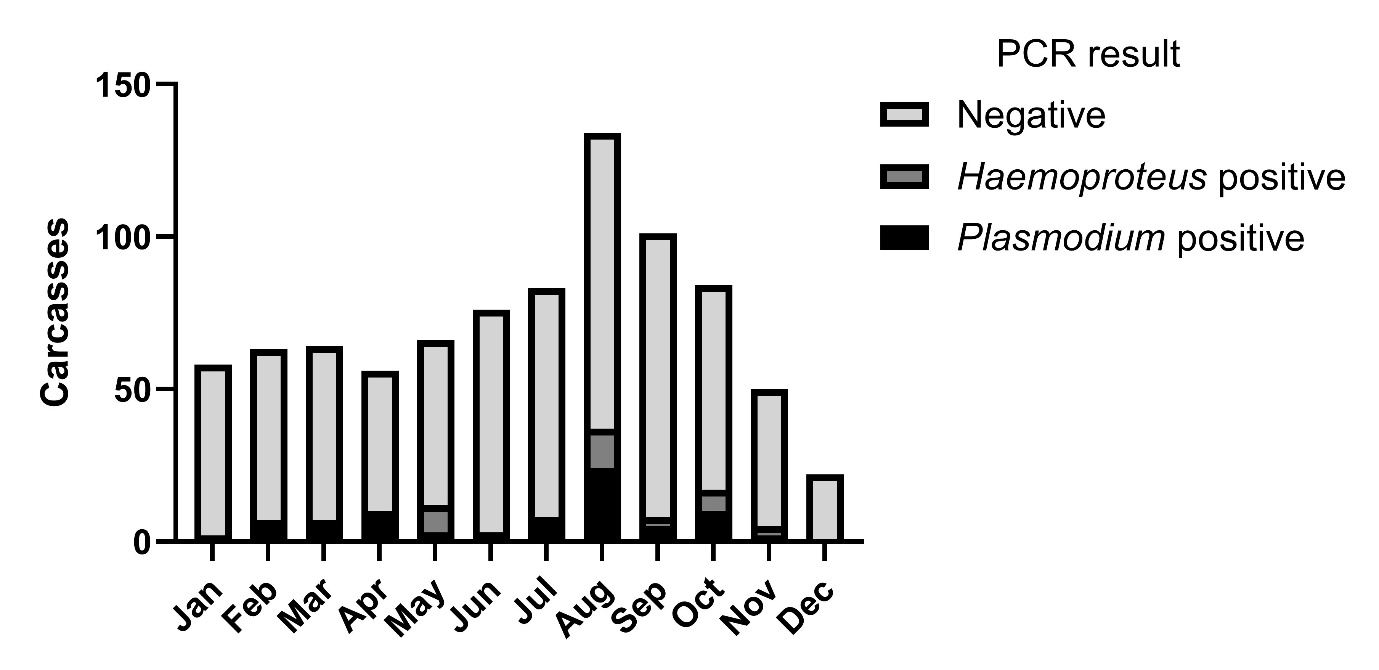
**

**Figure S3.** Infection rate of wild birds in Great Britain with avian haemosporidian parasites of the genera *Plasmodium* and *Haemoproteus* according to cause of death category (ascertained at post-mortem examination prior to molecular diagnostics).

**
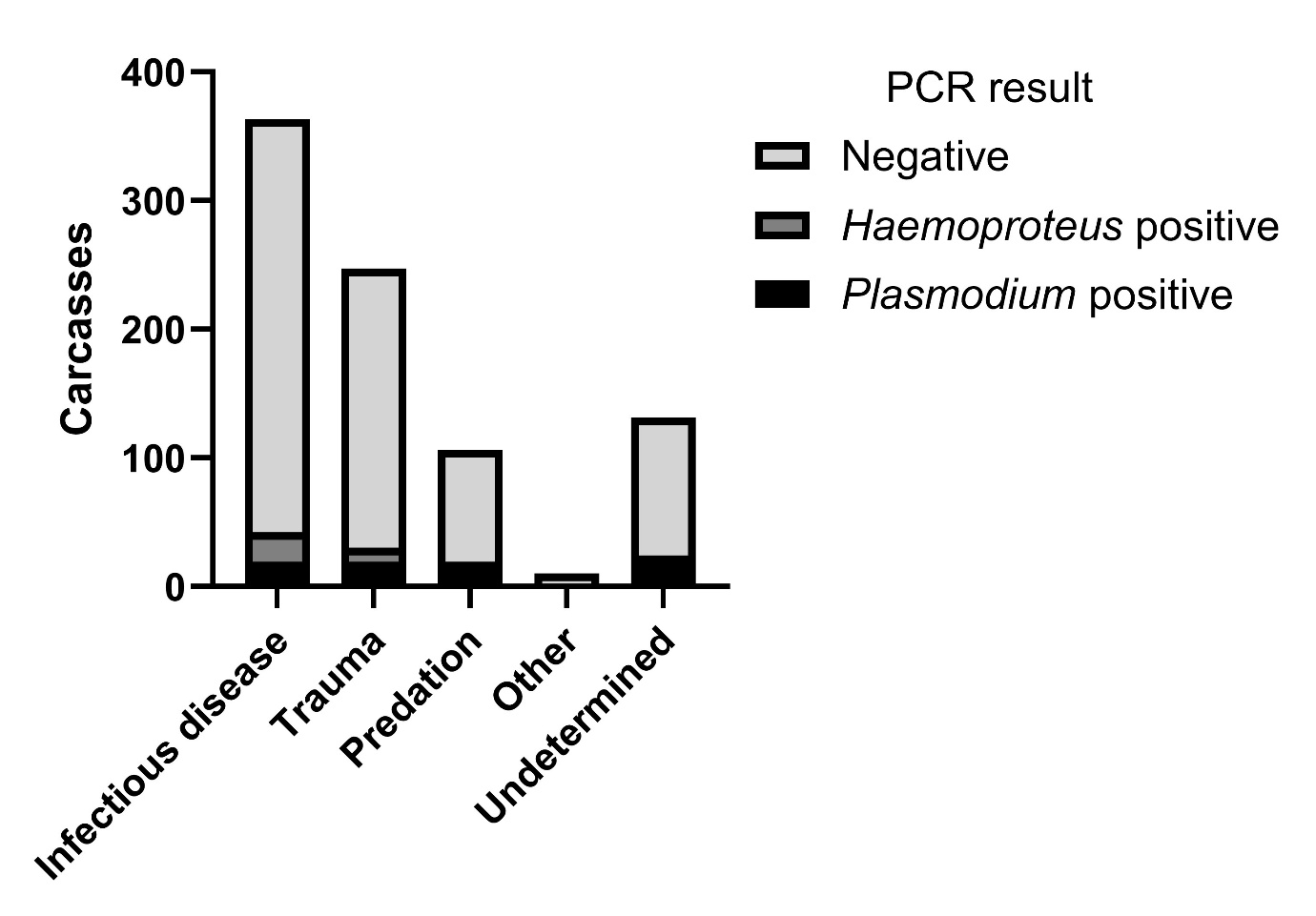
**

**Table S2.** Summary of histological findings in 13 *Plasmodium* sp. PCR-positive blackbirds (*Turdus merula*). * denotes individuals from which tissue samples concomitantly tested PCR positive for Usutu virus (Folly et al., 2020; Lawson et al., 2022). ** denotes individuals from which tissue samples concomitantly tested PCR positive for *Isospora* sp. (Yaffy et al., unpublished data). Organomegaly: H = hepatmegaly only; S = splenomegaly only; H+S = hepatosplenomegaly noted on gross post-mortem examination. Tissues examined: Lv = liver; Lg = lung; H = heart; K = kidney; S = spleen; Br = brain; M = skeletal muscle; Bs = bursa of Fabricius; P = pancreas; I = intestine. “Cause of Death Category” refers to cause of death attributed to each case based on post-mortem examination findings prior to molecular diagnostics being conducted.

| **Case ID** | **Signalment** | **Cause of Death category** | **Body condition** | **Organomegaly** | **Lineage** | **Tissues examined** | **Haemozoin** | **Meronts** | **Haemosiderosis** | **Malarial lesions** | |
| --- | --- | --- | --- | --- | --- | --- | --- | --- | --- | --- | --- |
|  |  |  |  |  |  |  |  |  |  | **Present** | **Comments** |
| XT0041-07* | Adult  Female | Infectious disease (systemic isosporiasias) | Emaciated | H+S | *Plasmodium* *matutinum* LINN1 | Lv; Lg; H; K; S; Br | **+** | **-** | **-** | **-** | Marked, multifocal, portal and random lymphoheterophilic necrotising hepatitis with marked sinus lymphocytosis. Diagnosed as systemic isosporiasis. |
| XT1174-07 | Adult  Male | Undetermined | Thin | S | *Plasmodium* *matutinum* LINN1 | Lv; Lg; H; S; Br | **+** | **-** | **+** | **+/-** | Marked, multifocal, heterophilic and lymphoplasmacytic interstitial pneumonia and vasculitis with rare apicomplexan meronts. Moderate, multifocal, portal and random lymphoheterophilic necrotising hepatitis with multifocal, intraparenchymal trematode eggs. |
| XT1189-07 | Adult  Unknown | Undetermined | Thin | H+S | *Plasmodium* *matutinum* LINN1 | Lv; H; S; Br; M | **-** | **-** | **-** | **-** | Marked congestion of liver and spleen. No evidence of hepatic or splenic necrosis. |
| XT0574-20 ** | Juvenile  Unknown | Infectious disease  (Usutu virus) | Normal | H+S | *Plasmodium* *matutinum* LINN1 | Lv; Lg; H; K; S; P; M; I; Br; Bs | **-** | **-** | **-** | **-** | Mild, spotty, portal to random lymphoplasmacytic and necrotising hepatitis. Mild, multifocal peri-ellipsoidal splenic necrosis. Diagnosed as USUV disease. |
| XT0962-05 | Adult  Male | Undetermined | Thin | H+S | *Plasmodium* sp. AFTRU5 | Lv; Lg; H; K; S; Bs | **+** | **-** | **+** | **-** | Splenic congestion. No evidence of hepatic or splenic necrosis. |
| XT0962-06 | Adult  Male | Undetermined | Thin | - | *Plasmodium* sp. AFTRU5 | Lv; Lg; H; K; S; Br | **+** | **-** | **+** | **+/-** | Mild, multifocal, random, lymphoplasmacytic hepatitis (possible chronic malaria). |
| XT0419-07 | Adult  Male | Infectious disease | Emaciated | - | *Plasmodium* *vaughani* SYAT05 | Lv; Lg; H; Br; M; G | **-** | **-** | **-** | **-** | Mild to moderate, multifocal, necrotising and lymphoplasmacytic portal hepatitis |
| XT0273-09 | Adult  Female | Predation | Normal | - | *Plasmodium* *vaughani* SYAT05 | Lv; Lg; H; K; S; Br | **-** | **+** | **-** | **+/-** | Mild, multifocal, random, lymphoplasmacytic and necrotising hepatitis |
| XT0145-13 | Adult  Male | Trauma | Normal | - | *Plasmodium* *vaughani* SYAT05 | Lg; H; K; S; Br; G | **-** | **-** | **-** | **-** | No diagnosis. |
| XT0911-13 | Juvenile  Male | Trauma | Normal | H | *Plasmodium* *vaughani* SYAT05 | Lv; Lg; S; Br | **+** | **+** | **+** | **-** | Rare apicomplexan meronts in pulmonary endothelial cells. Autolysis prohibited definitive diagnosis. |
| XT1018-14 | Juvenile  Male | Undetermined | Thin | H | *Plasmodium* *vaughani* SYAT05 | Lv; Lg; K; S; Br | **+** | **+** | **-** | **+** | Severe, focally extensive, necrotising heterophilic hepatitis and splenitis. Consistent with malaria. |
| XT0996-15 | Juvenile  Unknown | Undetermined | Thin | H | *Plasmodium* *vaughani* SYAT05 | Lv; Lg; H; K; S; Br | **+** | **+** | **+** | **+/-** | Mild, multifocal, random, lymphoplasmacytic hepatitis (possible chronic malaria). |
| XT0589-20 ** | Unknown | Trauma  (Usutu virus) | Normal | - | *Plasmodium* *vaughani* SYAT05 | Lv; Lg; H; K; S; P; M; I | **-** | **-** | **-** | **-** | Mild, spotty, periportal lymphoplasmacytic and necrotising hepatitis. Mild, multifocal peri-ellipsoidal lymphoplasmacytic and necrotising splenitis. Diagnosed as USUV disease. |
